# Factors Influencing Cage Escape Behavior in a Migrating Passerine at a Stop-Over Site

**DOI:** 10.3390/ani15131945

**Published:** 2025-07-01

**Authors:** Dariusz Jakubas, Katarzyna Wojczulanis-Jakubas, Marta Witkowska, Aleksandra Lesiewicz, Brygida Manikowska-Ślepowrońska, Izabela Wiśniowska, Łukasz J. Binkowski

**Affiliations:** 1 Department of Vertebrate Ecology and Zoology, Faculty of Biology, University of Gdańsk, 80-308 Gdańsk, Poland; biokwj@univ.gda.pl (K.W.-J.); m.witkowska@ug.edu.pl (M.W.); aa.lesiewicz@gmail.com (A.L.); biobms@ug.edu.pl (B.M.-Ś.); 2 Institute of Biology and Earth Sciences, University of the National Education Commission, Krakow, Podchorążych 2, 30-084 Krakow, Poland; wisniowska.izabela@doktorant.uken.krakow.pl (I.W.); lukasz.binkowski@uken.krakow.pl (Ł.J.B.)

**Keywords:** cognitive abilities, exploratory behavior, migratory birds, stop-over ecology

## Abstract

Cognitive abilities play a critical role for migratory birds encountering unfamiliar habitats at stop-over sites. We investigated factors (age, sex, fuel reserves, vertical/horizontal activity, and mercury level) affecting cognitive abilities behavior (problem-solving task—escaping from an experimental cage) in a long-distance migratory passerine, the sedge warbler (*Acrocephalus schoenobaenus*) at an autumn stop-over site. During the experiment, after two minutes of acclimatization, we remotely opened the door of the cage and recorded whether each bird escaped or remained in the cage. A mixed-effects Cox model revealed that the probability of escaping from the cage was significantly affected by variance in horizontal movements along the horizontal axis of the cage during the acclimatization phase (in models for all individuals and immatures) and sex (in immatures). Individuals moving more were more likely to escape from the cage. Immature males, at any given time, were 2.3 times more likely to escape from the cage than females. We interpreted this in the context of risk-partitioning with males adopting a high-risk, high-reward strategy and females using a low-risk, low-reward strategy, which may be associated with a predisposition for sex-specific roles in breeding. The repeatability of escape response was low (7.3%) for the same recaptured individuals.

## Simple Summary

Cognitive abilities play a critical role for migratory birds encountering unfamiliar habitats at stop-over sites. We investigated factors (age, sex, fuel reserves, activity in a cage, and level of mercury) affecting cognitive abilities and behavior (problem-solving task—escaping from an experimental cage) in a long-distance migratory passerine, the sedge warbler (*Acrocephalus schoenobaenus*) at an autumn stop-over site. In our experiment, after two minutes of acclimatization, we remotely opened the door of the cage and recorded whether each bird escaped or remained in the cage. Individuals moving more along the horizontal axis of the cage during the acclimatization phase were more likely to escape from the cage. Immature males were more likely to escape from the cage than females at any given time. We interpreted this result as risk-partitioning between sexes, with males behaving in a riskier manner (being more ready to escape) compared to females, which minimize predation risk. These differences may be linked to the predisposition of both sexes in the studied species to specific parental roles. We found that the repeatability of escape response after the cage door opened was low (7.3%) for the same recaptured individuals.

## 1. Introduction

Cognitive abilities, which allow animals to acquire, process and store information for use in the near or distant future, serve as an important component of animal adaptation to the environment (reviewed in [1,2,3]). It is generally hypothesized that natural selection plays an important role in generating local cognitive adaptations [4]. Multiple studies have demonstrated that exposure to a wide array of environmental stimuli enhances cognitive development, both at the inter-individual level (e.g., Ref. [5]) and across various taxa (e.g., Ref. [6]). However, other studies have shown that species living in environments of high variability tend to perform relatively poorly on cognitive tasks compared to species from stable environments, which would suggest that environmental variability may hamper, more than stimulate, cognition (e.g., Refs. [7,8]). This discrepancy is intriguing and highlights the complexity of the relationship between cognition and environment. To better understand cognitive-environmental relationships, further studies in diverse ecological groups are needed as many important ecological groups are greatly underrepresented.

In this context, the behavior of migratory birds offers an excellent study system. During the annual cycle, they are exposed to extraordinary environmental variability, which is related to the movements between breeding and non-breeding locations [9]. Seasonal migration, with an imposed habitat change, requires cognitive ability-linked traits such as a long-lasting memory and fast acquisition of information in unfamiliar environments. Additionally, the cognitive abilities of migratory birds are studied less frequently compared to other avian adaptations to migration (morphological, physiological, hormonal, and behavioral) [10]. Thus, investigating cognitive performance in a wild migratory species may not only enrich the area of cognitive ecology but also provide interesting insights into avian adaptations to seasonal migrations.

In this study, we examined the cage escape behavior of a wild avian species at a stop-over site during the autumn migration. The cage escape behavior experiment was performed to measure birds’ cognitive ability, defined as innovativeness (the ability to solve new problems) [11]. Each tested individual was required to solve a task (finding the exit) in order to get a reward (escaping from a novel, unknown place). Success in this problem-solving task is affected by various cognitive traits, like the perception of a change in environment (attention to the cage gate opening) and use of information (identifying the change as an opportunity to escape, making the decision about escaping). Thus, the cage escaping behavior is strongly linked to individual cognitive abilities [2,12]. The cage escape behavior is also driven by personality traits such as likeliness to explore and neophobia (an aversion to novelty) [13,14,15], as well as some other traits, like sex, age, and overall body condition. Sex and age are often considered potential factors regulating individual behavior in different contexts, as demonstrated in multiple studies (e.g., Refs. [16,17,18]). Thus, controlling for the effect of sex facilitates a better understanding of behavioral flexibility. Body condition (fuel reserves), health status (e.g., contamination level), and daily activity rhythm (hour of capturing) may also affect an individual’s motivation and overall performance (e.g., Refs. [19,20,21,22]) and are, therefore, important to be taken into account.

In this study, we used the sedge warbler (*Acrocephalus schoenobaenus*) as a model species. It is a small insectivorous passerine species, breeding in Eurasia and wintering in Central Africa [23]. During the autumn migration, it tends to fuel up extensively upon encountering a site with super-abundant prey (aphids or other aggregated invertebrates [24,25,26,27]) and subsequently flies directly to wintering grounds without refueling [28,29]. Our previous study compared the in-cage behavior of sedge warblers and closely related reed warblers (*Acrocephalus scirpaceus*). It revealed that by adopting different migration and refueling strategies at autumn stop-over sites (accumulating the amount of energy needed to fly safely from one stop-over site to the next and defending small, temporary territories at some stop-over sites to secure successful fueling), sedge warblers generally escaped sooner after the door was opened and were 1.79 times more likely to escape, at any given time, than reed warblers [30]. Thus, in this study, we focused solely on the sedge warbler and factors affecting escape probability at the intra-species level.

We investigated the cage escape behavior of the sedge warbler in relation to age, sex, and some individual characteristics related to personality (activity in the cage), body condition (body mass and fat score), contamination status (mercury level in feathers), and activity rhythm (hour of capture). For this purpose, we formulated the following expectations:

(1) Given age differences in migratory experience (immatures migrate for the first time in their life) and spatial neophobia (immatures may be less eager to explore new environments than adults as they have less experience to compare new situations with known ones [31]), we expected that adults would escape from the cage more frequently than immatures.
(2) Given sex differences in cognitive and learning abilities and risk-taking behavior [16,17,18], we expected higher activity levels in males, resulting in a higher probability of their escape from the cage.
(3) Given various motivations for foraging of individuals with different fuel reserves [19,20] and the results of the previous study showing that lean individuals of the same species explored an unfamiliar environment (cage) at a stop-over site more than fat birds [20], we expected that individuals with lower body conditions (body mass and fat score) to be more motivated and, consequently, escape more frequently compared to individuals with higher fuel loads.
(4) Given that exploratory behavior in birds is positively correlated with overall activity, risk-taking behavior, boldness towards novel objects, and aggressiveness [32,33], we expected individuals with a higher activity level to be more probable to escape.
(5) Given the negative effects of mercury (Hg) contamination on birds (summarized in [34,35]) and especially its potential effects on the physiological aspects of fattening, it is possible that Hg exposure could cause behavioral changes that would reduce a migrant’s stop-over refueling performance (summarized in [36]). For example, high Hg levels have been observed to suppress the appetite and the motivation to forage in birds [21,22]. High Hg levels have also been associated with lethargy and ataxia (summarized in [36]). Consequently, we expected individuals with higher Hg concentration to escape from the cage less often than those with lower levels.
(6) Given that the daily activity of birds can change over time of the day (following the activity of insects and other ectothermic prey) and that fuel reserves can be lower after the night (due to the capturing of incoming migrants with low fuel reserves, as the sedge warbler is a nocturnal migrant [23]), we expected that individuals captured and tested in the morning hours would behave differently and, in turn, exhibit an inconsistent probability of escaping from the cage. It is hard to predict the direction of differences, as birds captured in the morning hours may be more motivated to refuel, being unable to forage just after landing (as newly arrived birds also had a slightly reduced capacity for food absorption because of significant losses to digestive organs, which may serve as a reservoir of protein catabolized for fuel during flight [37,38]).

## 2. Materials and Methods

### 2.1. Study Area

We conducted our study in the southern part of the Lake Druzno Reserve (54°05′ N, 19°27′ E) in Northern Poland, during the annual autumn ringing of small passerines in 2021 and 2022. Lake Druzno is a large, shallow eutrophic lake, overgrown with reedbeds over much of its area, making it an attractive breeding and stop-over site during spring and autumn migrations for sedge warblers and other reedbed-associated passerines [39,40].

### 2.2. Trapping of Birds

We captured birds in reedbeds in August (covering almost the entire autumn migration period of birds from the genus *Acrocephalus* in this area [39]) from dawn to dusk in mist-nets with a mid-day brake during the hottest part of the day (between 11:00 and 16:00). Caught individuals were ringed and aged as adults or immatures (i.e., hatched in the same calendar year) based on external characters [41]. We measured wing length (flattened) with a ruler (±1 mm) and body mass using an Ohaus CL 201 electronic scale (accurate to 0.1 g) (Ohaus, Parsippany, NJ, USA). To estimate fat reserves, we determined the fat score on a 0–8 scale [42]. From each immature individual, we collected a few body feathers from its back for molecular sexing and mercury level analyses (see details in the next subchapters). We did not sample feathers from adults, expecting a small sample size for this age group a priori; dividing this age group into sexes would preclude statistical analyses.

### 2.3. Cage Escape Experiment

We performed cage experiments between 10–24 August in 2021 and 7–19 August in 2022, during the core of the migration period for the studied species at the study site, i.e., excluding the period in turn of July and August with a potentially large proportion of local individuals not yet on migration [39,43,44]. Another study conducted in the same study area has revealed that 97% of sedge warblers tested during the autumn migration (1 August–15 September) in the orientation cage showed significantly oriented behavior [45], suggesting high levels of migratory restlessness in the individuals tested during this period.

In the cage experiments, we used the setup previously described in [30]. Immediately after ringing and measuring, we placed each individual into an experimental cage (commercial type; dimensions: 52.5 cm × 32.5 cm × 72.0 cm). The cage was equipped with two horizontally placed perches located on both sides of the entrance door (dimension: 13.0 cm × 13.0 cm), located in the middle of the front wall. We covered every side of the cage except for the front one with the door to minimize the impacts of external stimuli on behavior of tested individuals (see cage photos and videos from the experiment in [30]). We attached a few-meter-long string to the cage door, allowing for remote opening without direct manipulation during the experiment. The experimenter was not visible to the tested bird. The whole experiment lasted a total of four minutes, consisting of two phases. In the first acclimatization phase, lasting one minute, an individual was simply put into the closed cage to adjust to the new environment. The last 30 s of this phase were also used for measuring the activity level. In the second phase of the experiment, we opened the door remotely by pulling the string, allowing the bird to spontaneously escape from the cage within three minutes. If that did not happen, we released the bird. We filmed all the experiments using a commercial camera (Panasonic HC-V180) setup at a distance of 0.5 m from the cage. The videos were then manually processed in VLC media player software ver. 3.0.20 (VideoLan, Paris, France, https://www.videolan.org/vlc/index.html, accessed on 27 June 2024) to establish the occurrence and the timing of the spontaneous escape of the individual. The videos were also used for birds’ movement analysis (see details in Section 2.4). Cage escape experiments were performed from 06:00 to 20:00 by A.L. and M.W.

In total, we performed 258 cage tests (124 in 2021 and 134 in 2022). Within those tests, we conducted 239 tests on immatures (92 on females, 139 on males, and 4 on unsexed individuals) and 19 on adults (unsexed individuals). We tested 236 individuals (with 16 recaptured immatures being tested > 1), 217 immatures (87 females, 127 males, and 3 unsexed individuals) and 19 adults (all unsexed individuals).

### 2.4. Analysis of Bird Movement in the Cage

To measure bird activity in the cage, we traced, on the videos, all the bird’s movements during the last 30 s of the acclimatization phase before the opening of the door. All the birds’ stationary locations (i.e., between changes of location by flying or hopping) were annotated manually in the two-dimensional space (x and y axes), using Tracker software ver. 6.0.9 (https://opensourcephysics.github.io/tracker-website/, accessed on 27 June 2024), after distance calibration (setting the scale and the angle). Using the Pythagorean theorem and trigonometric functions, we calculated the distance between each pair of stationary points and later summed these distances as the total distance covered in the cage during the last 30 s of the acclimatization phase. We assumed here that a longer distance covered by an individual indicated a higher activity level. We also calculated the variance in stationary locations, considering the x and y axes separately. Variance values reflected the intensity of bird movements along the cage’s horizontal (x, hereafter referred to horizontal movement variance) and vertical (y, hereafter referred to vertical movement variance) axes. We assumed that higher variance of horizontal and/or vertical movements indicated higher motoric activity. Tracking of individual movements was performed by A.L.

### 2.5. Sexing of Captured Birds

Constrained by the sample size, we sexed only immature individuals. To sex them, we used a combination of morphological and molecular techniques. For this purpose, we used a discriminant function based on individuals captured during autumn migration in the studied area [46]. This function includes flattened wing length and allows for 83% correctness in sex identification [46]. Based on this function, we directly assigned sex to individuals with wing lengths in ranges with a >90% probability of correct identification (i.e., wing length of <65 mm (females) or >67 mm (males) [46]). Individuals with an intermediate wing length (sexed with a ≤90% probability of correct sex identification [46]) were then sexed molecularly. For this, we extracted DNA from feather samples, using the Sherlock AX (A&A Biotechnology, Gdynia, Poland). We then followed the method in [47] to perform polymerase chain reactions, using primer pair P2 and P8 as well as 50 °C for the annealing temperature. The primer pair amplified introns on the CHD-W and CDH-Z genes located on the W and Z avian sex chromosomes that varied in length [47]. The difference between the two fragments (~20 bp) was clearly visible in UV light when separated on 2% agarose gel and stained with Midori Green. Molecular sexing was performed by M.W. in the laboratory of the Department of Vertebrate Ecology and Zoology in the Faculty of Biology at the University of Gdańsk, Poland. We successfully sexed 99% of immature individuals.

### 2.6. Mercury Concentration in Feathers

We analyzed mercury (Hg) levels in the body feathers of the sedge warbler. As for sexing, given the small sample size of adults, we analyzed mercury levels only in immature individuals. Since it has been found that concentrations of Hg in blood and feathers are strongly correlated in birds, the keratinous tissues chosen in this study are relevant for a survey of Hg exposure [48].

We washed feathers prior to analysis. We then transferred samples to tubes containing 10 mL of a chloroform–methanol mixture (2:1, *v*/*v*; both reagents from Chempur, Poland) and placed them in an ultrasonic cleaner (Ultrasonic Cleaner USC-THD, VWR, Radnor, PA, USA) for 5 min. Next, we transferred the samples to 50 mL Falcon tubes filled with 10 mL of methanol and placed them on an orbital shaker (SK-03330-PRO, Chemland, Poland) for 1 min. Subsequently, we transferred the samples into watch glasses and left them to dry for 48 h. Finally, we homogenized the dried samples (by cutting them into a fine powder using scissors) and stored them in Eppendorf-type tubes.

We measured total Hg concentration in feathers with a cold vapor atomic absorption spectrometer (Nippon Instrument Corporation, MA-3, Kyoto, Japan). Depending on the sample size, we usually used 0.5 mg (0.1 mg–1.6 mg) of dry-weight (d.w.) feather, with no prior mineralization. We burned the organic matrix at a temperature of 850 °C, bonded vapors of Hg by amalgamation on a gold trap, fried Hg from the amalgamate by heating and measured the absorption at 253.7 nm. In order to minimize potential interference, we supplemented each sample with additive B (Wako Pure Chemical Industries Ltd., Osaka, Japan). We performed each analysis at least twice for each sample. If RSD was lower than 10%, we used the mean of the measurements as the final result. Otherwise, we reanalyzed the sample. We expressed final results in the unit of μg/g d.w. We checked the whole procedure against the certified reference material (CRM)—lobster hepatopancreas TORT-3 (NRC, Ottawa, ON, Canada; certified Hg concentration: 0.292 ± 0.022 μg/g d.w.). We analyzed the CRM samples at the beginning and end of each analytical cycle. We adjusted the weight of the CRM to represent an amount of Hg similar to that in feathers. Our measured values for the CRM were 0.305 ± 0.03 μg/g d.w. (n = 27), showing a recovery of 104.59 ± 8.99%. The detection limit was 0.03 ng per sample. Mercury concentration analysis was performed by I.W. and Ł.J.B. in the laboratory at the Institute of Biology and Earth Sciences of the University of the National Education Commission, Krakow in Kraków, Poland.

### 2.7. Statistical Analyses

We performed all statistical analyses in R software ver. 4.4.2 [49]. To assess statistical significance throughout, we used α = 0.05 as the level of significance. As the experimental setting was the same over the whole study period (including the placement of the experimental cage and camera), we combined data from both years in all of the analyses. The small sample size in studied years, especially for adults, further supports our decision to pool our data. Since we performed sexing and mercury (Hg) concentration analyses exclusively in immatures, we performed two separate sets of analyses. The first set of analyses included the results of the cage experiment for all individuals, i.e., adults (N = 19) and immatures (N = 217; hereafter referred to collectively as both age groups), and we used it primarily to examine the effect of age on cage escape behavior. The second set included the results of the cage experiment for immatures only (N = 87 females, N = 127 males; hereafter referred to as the immatures group) and contained two additional variables: sex and Hg concentration.

Firstly, we compared the proportion of individuals that escaped and did not escape from the experimental cage between age (for both age groups) and sex (for the immature group) groups using either a chi-square test of independence or Fisher’s exact test. Then, we calculated the odds ratio for escaping the cage. We calculated the chi-square test, Fisher’s exact test, and odds ratio using the *rcompanion* package ver. 2.4.36 [50]. Because of a relatively small sample size for adults, we calculated the power of the Fisher’s exact test by simulating the proportions of immatures and adults that escaped from the cage using the *power.fisher.test* function from the *statmod* package ver. 1.5.0 [51].

Secondly, to examine the probability of escape from the cage with respect to time, we used mixed-effects Cox models with the number of seconds it took the bird to escape as the response variable. For the model using data from both age groups, we set age as the factorial explaining variable, then fat score, body mass, total distance covered in the cage, horizontal movement variance, vertical movement variance, and capturing hour as continuous explanatory variables, and bird identity (ring number) as a frailty (i.e., a random effect; the frailty function allows adding a single random effects term to a Cox model). For the model with data from immatures only, we used the following set of explanatory variables: sex as a factorial effect, fat score, body mass, total covered in the cage, horizontal movement variance, vertical movement variance, capturing hour, and Hg concentration of feathers as continuous explanatory variables, with bird identity (ring number) as a frailty (random effect). We performed mixed-effects Cox models using the *coxph* function in the *survival* package ver. 3.8.3 [52]. To avoid overfitting the models, we included only main effects, omitting the interaction terms.

In both models, we considered the birds that did not escape within three minutes as ‘censored’ observations (i.e., they were still in the dataset when the observation time expired but had not yet escaped). The hazard function for each group represents the likelihood that a given bird will escape the cage at a given time, assuming it is still in the cage at that time [53]. We checked the Cox proportional hazards model assumption that the hazard functions of the two groups are proportional across time using the *cox.zph* function in the *survival* package ver. 3.8.3 [52]. We also checked multicollinearity using the *vif* function in the *rms* package ver. 8.0-0 [54].

We visualized the probability of birds staying in the cage using a Kaplan−Meier plot of the survival curves using the *surviminer* package ver. 0.5.0 [53]. We estimated counterfactual survival curves for specific values of a continuous covariate using the *contsurvplot* package ver. 0.2.1 [55] and visualized them in the *ggplot2* package ver. 3.5.1 [56].

Since we did not include interaction terms in our models, we further investigated sex as a potential predictor significantly affecting the probability of escape from the cage at a given time in the immature group (i.e., movements along the horizontal axis of the cage). For this, we used a mixed permutational analysis of variance (MPANOVA) with 5000 permutations with bird identity (ring number) as a random factor in the *permuco* package ver. 1.1.3 [57]. We also used MPANOVA to compare Hg concentrations between immature males and females.

Finally, given that we performed experiments more than once, in the case of 16 recaptured immatures (6 females, 9 males, and 1 unsexed), we also investigated whether escape response was repeatable in subsequent trials for the same individuals. To this end, we used repeatability analysis as implemented in the *rptR* package ver. 0.9.22 [58]. For the repeatability analysis, we employed a generalized linear mixed model with the binomial family with bird identity (ring number) as a random factor.

## 3. Results

### 3.1. Proportions of Individuals That Escaped from the Cage

Although adults tended to escape from the cage more often (63%; N = 19) than immatures (42%; N = 239 tests), this difference was not statistically significant (Fisher’s exact test; *p* = 0.092) and the power of the test was relatively low (0.36).

When considering only sexed immatures (N = 235 tests), we found that males escaped from the cage more frequently (50%; N = 139 tests) than females (31%; N = 96 tests; chi-square test of independence, χ^2^_1_ = 7.877, *p* = 0.007). The estimated odds ratio for this difference was 2.17 (with a 95% confidence interval of 1.26–3.74), indicating that immature males were 2.17 times more likely to escape from the cage than immature females.

### 3.2. Factors Affecting Cage Escape Probability

#### 3.2.1. Both Age Groups

**Figure 1:**
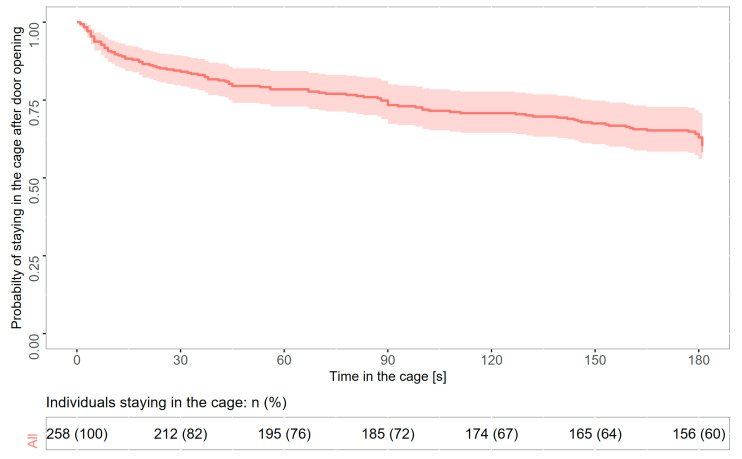
Probability of staying in the cage expressed as Kaplan−Meier survival curves for both age groups of sedge warblers tested during the autumn migration at the stop-over site. The shaded area represents 95% confidence intervals.

**Figure 2:**
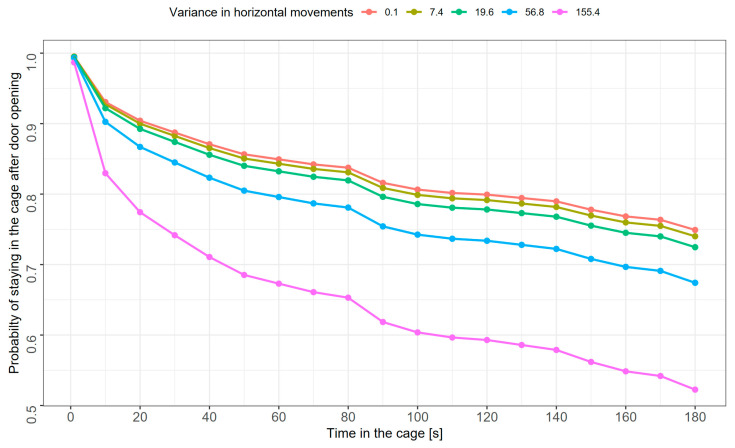
Curves of estimated probabilities of staying in the cage after door-opening in both age groups of sedge warblers for various levels of horizontal activity expressed as variance of movements along the horizontal axis of the cage. Presented levels represent the 10th, 30th, 50th, 70th, and 90th percentile values of variance in horizontal movements (from left to right in the legend).

A mixed-effects Cox model revealed that among the studied variables, only the variance in horizontal movements significantly affected the probability of escaping from the cage (Table 1). Kaplan−Meier survival curves for all individuals revealed that after 30 s with the cage door open, 82.2% (212 of 258 tests) of tested sedge warblers were still in the cage. At the end of the experiment (i.e., 180 s after the cage door opening), 60.5% of sedge warblers were still in the cage (Figure 1). Individuals moving more along the horizontal axis were more likely to escape from the cage (Figure 2). The model assumption about proportional hazards (proportional across time) was upheld (global *p* = 1.0; *p* for particular predictors: 0.07–0.57).

#### 3.2.2. Immatures

**Figure 3:**
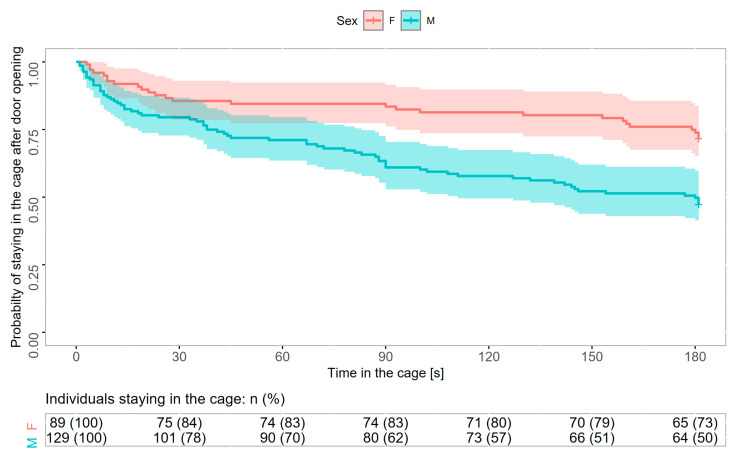
Probability of staying in the cage expressed as Kaplan−Meier survival curves for immature females (F, red) and males (M, blue) of sedge warblers tested during the autumn migration at the stop-over site. The shaded area represents 95% confidence intervals.

**Figure 4:**
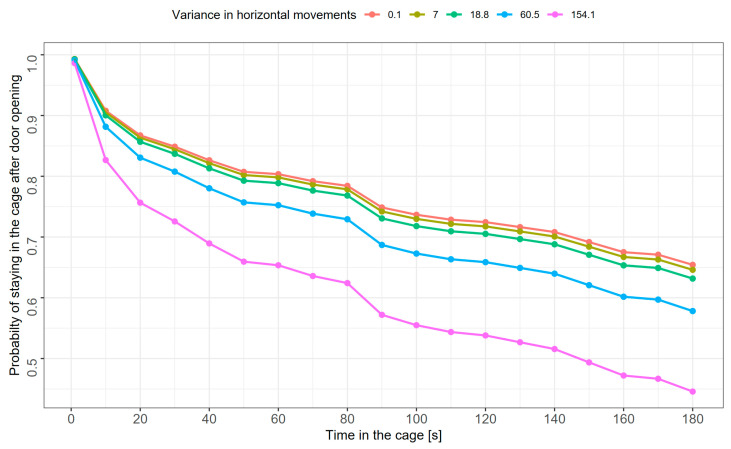
Estimated probability curves of staying in the cage after opening the door for immature sedge warblers (both sexes combined) with various levels of horizontal activity, expressed as the variance of movements along the cage’s horizontal axis. Presented levels represent the 10th, 30th, 50th, 70th, and 90th percentile values of variance in horizontal movements (from left to right in the legend).

The mixed-effects Cox model considering only immatures revealed that the probability of escaping from the cage was associated with sex and variance in horizontal movements (Table 2). There was a significant difference in hazard functions between males and females, as expressed by the hazard ratio of 2.27 (95% CI: 0.81–3.66), indicating that at any given time, males were 2.3 times more likely to escape from the cage compared to females (Figure 3). Kaplan−Meier survival curves for all individuals revealed that after 30 s with the cage door open, 84.3% (75 of 89 tested) of females and 78.3% (101 of 129 tested) of males were still in the cage. At the end of the experiment (i.e., 180 s after the cage door opening), 73.0% of females and 49.6% of males were still in the cage (Figure 3). Individuals moving along the horizontal axis had more had a higher probability of escaping the cage (Figure 4). The model’s assumption about proportional hazards (proportional across time) was upheld (global *p* = 0.73; *p* for particular predictors: 0.10–0.92).

To check whether the variance of movements along the horizontal axis of the cage was affected by sex, we compared horizontal movement variance between females (mean: 45.2) and males (mean: 56.6). We did not find a significant difference (MPANOVA; F = 1.319; resampled *p* = 0.248).

### 3.3. Repeatability of the Cage Escape Behavior in Immature Individuals

We found that the escape response in the same recaptured individuals was not significantly repeatable (R = 0.073, SE = 0.133, CI: 0–0.472, *p* = 0.339; N = 16 recaptured individuals tested more than once).

## 4. Discussion

We found that the proportion of immature sedge warblers that escaped and did not escape from the cage (42% and 58%, respectively; N = 239) was comparable to the results of a similar experiment performed on the same species at same site and migration phase in previous years (in 2016, 2017, and 2019) (41% vs. 59%; N = 93; χ^2^ test with Yates’ correction, χ^2^_1_ = 0.001, *p* = 0.969) [30], indicating pattern consistency among the years. The relatively low number of individuals that escaped from the cage (<50% in both cases) may be explained by the fact that migratory species like the sedge warbler are more likely to enter an unfamiliar environment but are less explorative once there when compared to resident species [14,59]. Migrants spending relatively short periods of time at each stop-over site must quickly familiarize themselves with local food resources and predation risk [60]. Studies on migratory passerines have indicated that predator avoidance is a priority at stop-over sites and that predator avoidance behavior may limit foraging areas to those which are safe from predation [61]. Given the benefits and risks of exploring an unfamiliar environment, exploration is expected to be kept to a minimum, as the gathered information cannot be used for long [62,63].

Contrary to our expectations, we did not find age differences in the cage escape behavior. This might be explained by the small sample size of examined adults (N = 19 individuals) compared to immatures (N = 217 individuals) and low power of the performed test (36%). It can also be associated with lower competition between age groups in the sedge warbler, as this species is not territorial during migration, focusing on highly abundant food. This is in contrast to a closely related but more competitive species, the reed warbler, which maintains territories even at stop-over sites [24,25,30]. In another migrating passerine species, the Wilson’s warbler (*Wilsonia pusilla*), some age differences in the pace of exploration and neophobia have been detected—migrating immatures showed longer exploratory movements during stop-over than adults. Such results were interpreted as the effects of a subordinate social status of juveniles and their lower efficiency in finding resources [64].

As we expected, individuals with higher activity levels in the cage (along the horizontal axis) during the acclimatization phase were more likely to escape at any moment after the door opened. Movement activity in an unfamiliar habitat or novel conditions is linked to risk-taking. It may be associated with higher mortality risk because of predation and/or parasite infection [65,66]. The risks associated with exploratory behavior vary according to local environmental conditions [67,68]. The cage is an artificial environment where the birds were kept for a short period of time, with no food and no apparent predators present. Thus, it is hard to interpret the escape behavior in the context of environment exploration and risk-taking strategies. On the other hand, we observed some inter-individual variation in the level of activity, suggesting that it may be a personality-linked trait. If this is the case, then the escape behavior would be a proxy for the bird’s overall performance in a novel environment. More exploratory individuals usually enhance their chances of survival by discovering new resources or territories and decreasing competition [69,70].

As we expected, we found sex differences in the probability of escaping from the cage in immature individuals. Although expected, the causality of the pattern is not straightforward to interpret. Behavioral sex differences are often explained in the context of sex differences in corticosterone and/or testosterone concentration [71], but recent studies have indicated that this is the case only during the breeding period. For example, levels of both hormones during the post-breeding period have been similar in a long-distance passerine migrant, the barn swallow (*Hirundo rustica*) [72]. Thus, sex differences recorded in the present study may be interpreted in the context of sex differences in risk-partitioning, when one of the sexes chooses a high-risk, high-reward strategy and the other a low-risk, low-reward strategy. Such partitioning has been described, e.g., in the context of sex differences in foraging strategies in seabirds, which allow for maximizing the food delivered to offspring across variable foraging conditions [73]. In the context of the present study, males might have adopted a high-risk, high-reward strategy, trying to escape the cage, while female behavior represents a low-risk but also low-reward strategy. Some studies (e.g., Ref. [74]) have perceived the cage escape response in the context of predation risk; birds that escaped more quickly or moved more in the cage may be more reactive and quicker to flee from predators. In this perspective, sex differences in the cage escape behavior may be interpreted in terms of sex-specific predation risk. For example, females of two waders, least sandpipers (*Calidris minutilla*) and western sandpipers (*Calidris mauri*), exhibited greater escape performance when released from the box after ringing compared to males. This suggests greater escape ability in females at greater risk of predation [75]. Thus, sexes may differ in how they process and later use information gathered during cage exploration and subsequent decision-making. The difference found in our study may also be interpreted in terms of behavioral predispositions for sex-specific roles in breeding. Since in sedge warblers only females incubate the eggs [23] and male contribution to brood care has been classified as reduced [76], one may expect different reactions to novel situations (including those associated with predation risk). Females might take less risk (e.g., staying motionless after detecting an approaching predator when incubating in the nest) compared to males (e.g., actively guarding the mate and competing with other males for territories and/or extra-pair copulations). Our study was performed during migration, and we investigated sex differences only in immatures. Nonetheless, if our interpretation of the mechanisms of the sex differences is correct, we believe that the presumed behavioral predisposition for sex-specific roles in breeding could also be expressed during other phases of an annual avian cycle, including migration. A separate study investigating this issue would be valuable. The sex differences in the cage escape response observed in our study may be also interpreted in terms of sex-specific neophobia. Studies on two small passerines, collared flycatcher (*Ficedula albicollis*) and pied flycatcher (*Ficedula hypoleuca*), have found that females were more neophobic towards novel objects in the nest [77,78].

In contrast to our expectation, we did not find a significant effect of body reserves (expressed as fat score or body mass) on the probability of cage escape, which is in contrast to other findings. For example, a study on blue tits (*Cyanistes caeruleus*) has revealed that lean birds explored novel objects in the experimental cage earlier than birds with higher fat scores [19]. Moreover, another experimental study performed on migrating sedge warblers found that lean individuals explored an unfamiliar environment (a cage with food) more than fat birds [20]. We explain the lack of a significant effect of body reserves in our study by a small variability in fat scores among the tested individuals, with a predominance of lean individuals (fat scores 0 and 1) constituting 87% of tested immatures and 79% of tested adults. Low fuel reserves at stop-over sites in autumn in this part of Europe have been reported in other studies (e.g., Refs. [39,79]) and have been explained by the gathering of larger reserves closer to barriers like the Mediterranean Sea and Sahara [79]. The strategy of gaining fuel reserves is also time-dependent. It has been found that early-migrating sedge warblers behaved as if they expected aphid peak conditions at succeeding stop-over sites, while late-migrating individuals, being too late for the aphid peak, seemed to expect less favorable feeding conditions at succeeding stop-over sites and thus stayed to exploit the local food surplus to reach large fuel loads at departure [80].

In contrast to our expectations, we did not find an effect of Hg concentration in feathers on the escape response of immature individuals tested in the cage. This result can be explained by the relatively low level of mercury in the feathers of the studied individuals, ranging from 0.24 to 5.99 µg/g d.w. (mean ± SD: 1.24 ± 0.85 µg/g d.w. for 81 females and 1.18 ± 0.70 µg/g d.w. for 120 males; no sex differences MPANOVA, F = 0.332, *p* = 0.576). A few Hg toxicity reference values have been proposed for avian feathers (summarized in Table 3). The majority of the studied individuals (91–100%, depending on the threshold) did not exceed the Hg toxicity threshold (Table 3). The highest Hg concentration, slightly exceeded two of the three thresholds, was found in one female with concentration values of 5.99 µg/g d.w.

We found that the repeatability of the escape response of recaptured immature individuals, tested more than once during a single year, was low (only 7%) and not statistically significant. This result may be explained by the fact that the same individuals may have different motivation levels in consecutive experimental trials [19,20].

We are aware of several limitations of the study. Firstly, in the experimental conditions, the tested birds might not express their natural behavior, as they could be simply frightened by the artificial conditions. However, our observations and results of the previous study with the same experimental settings [30] indicate that the majority of birds, after a few-second-long initial ‘hyperactive’ phase calmed down, indicating their acclimatization to the novel environment. They then started to explore the cage, hopping between the perches and sides of the cage or just sitting on the perch while observing their surroundings. Thus, we believe that both sex- and individual-specific behaviors should be expressed even in unusual conditions (experimental cage) and after a stressful situation (handling). Secondly, results for both age groups should be treated with caution, as we based our study on a small number of examined adults (N = 19 individuals). In addition, the repeatability of the escape response was based on a small sample size (N = 16 individuals). Small sample size can reduce the ability to detect statistically significant effects, leading to low statistical power. Thirdly, some of the studied variables (fat score and Hg concentration in feathers) were characterized by low values and variability, which reduce the power of a statistical test to detect a significant effect. Fourthly, there are many potential factors affecting the escape response of studied birds that we were not able to control for or did not analyze, including health status and time spent by birds in the mist net before disentangling. Finally, our interpretation of sex and inter-individual differences in the escape behavior, even if plausible, may not be necessarily related to the attributed differences in cognition-linked traits and/or risk-partitioning. More studies including various cognitive abilities traits in birds are needed to fully understand the factors affecting avian decisions about escaping/not escaping an experimental cage. Nevertheless, the observed inter-sex and inter-individual differences indicate various levels of success in the problem-solving task.

## 5. Conclusions

We found that the cage escape behavior in migrating sedge warblers was individual-dependent, as it was affected by the horizontal activity of the experimental individuals, regardless of sex (and also age). We also observed sex differences in immatures, with males being more likely to escape from the cage than females. This may be interpreted in the context of different risk-partitioning strategies, where the higher probability of escape in males could be attributed to adopting a high-risk, high-reward strategy, compared to females. We also found that Hg concentrations in the feathers of immatures were low, which likely explains the lack of a relationship between cage escape behavior and the concentration of this neurotoxic element.

Understanding the links between personality traits (e.g., exploratory behavior, neophobia) and cognitive abilities (e.g., problem-solving) is important for comprehending the reactions of migratory birds to rapidly changing environments. This, in turn, could help predict their reactions to global changes at stop-over sites. Migratory birds are threatened by multiple independent risks from global change, including climate change and anthropogenic changes in habitat quality and quantity (e.g., Refs. [83,84,85]). In the case of many species migrating along and staging in the coastal zone (including the studied species), expected global sea rise [86] may eliminate their traditional stop-over sites [87]. Their cognitive abilities may decide how they will adapt to this new situation.

## Figures and Tables

**Table 1:** Results of a mixed-effects Cox model for both age groups, with the probability of escape from the cage as the response variable.

Variable	HR ^1^	95% CI ^2^	*p*-Value
Age			
Immatures	—	—	
Adults	1.39	0.60, 3.24	0.445
Fat score	0.98	0.74, 1.28	0.864
Body mass	1.18	0.88, 1.57	0.268
Total distance covered	0.99	0.95, 1.03	0.660
Horizontal variance	1.01	1.00, 1.01	<0.001
Vertical variance	1.00	1.00, 1.00	0.228
Capturing hour	1.05	0.94, 1.17	0.348
Frailty (bird identity)			0.084

^1^, hazard ratio; ^2^, confidence interval.

**Table 2:** Results of a mixed-effects Cox model for immatures with the probability of escaping from the cage as the response variable.

Variable	HR ^1^	95% CI ^2^	*p*-Value
Sex			
Female	—	—	
Male	2.27	1.41, 3.66	<0.001
Fat score	1.02	0.81, 1.29	0.841
Body mass	0.92	0.70, 1.21	0.554
Total distance covered	0.99	0.96, 1.03	0.718
Horizontal variance	1.00	1.00, 1.01	<0.001
Vertical variance	1.00	1.0, 1.00	0.135
Capturing hour	1.09	0.99, 1.19	0.066
Hg concentration	1.01	0.77, 1.32	0.951
Frailty (bird identity)			0.756

^1^, hazard ratio; ^2^, confidence interval.

**Table 3:** Concentration of mercury (Hg) in the feathers of studied immature sedge warblers in relation to the mercury toxicity threshold proposed for feathers by [81,82].

Norm of Hg Concentration	Norm	Threshold Value	Individuals: n (%)	
(Reference)		[µg/g d.w.]	Below Threshold	Above Threshold
feathers [81]		5.0	200 (99.5)	1 (0.50)
EC * breast feathers [82]	EC1 **	2.3	184 (91.5)	17 (8.5)
	EC5 ***	5.7	200 (99.5)	1 (0.5)
EC * head feathers [82]	EC1 **	2.6	191 (95.0)	10 (5.0)
	EC5 ***	6.5	201 (100)	0 (0)

*—effective concentrations (EC) for Hg toxicity, i.e., effective Hg concentration at which the control-normalized response declined; it is expressed as percentage decline (injury) in the measured endpoint relative to the control: **—1% (low injury); ***—5% (moderate injury) [82].

## Data Availability

The dataset supporting this article has been uploaded as part of the electronic Supplementary Material.

## Supplementary Materials

The following supporting information can be downloaded at: https://www.mdpi.com/article/10.3390/ani15131945/s1, File S1: Supplementary data file with results of all experiments.
